# Application and clinical significance of tissue ultrasound for assessment of right ventricular diastolic function in patients with coronary slow flow

**DOI:** 10.12669/pjms.38.4.5342

**Published:** 2022

**Authors:** Xuefang Zhu, Xiuping Xu, Zhen Wei, Zenghua Zhu

**Affiliations:** 1 Xuefang Zhu, Department of Ultrasound, the third Hospital of Quzhou, 226 Baiyun North Avenue, Quzhou 324000, Zhejiang Province, P.R. China; 2 Xiuping Xu Department of the 3^rd^ Wards, the third Hospital of Quzhou, 226 Baiyun North Avenue, Quzhou 324000, Zhejiang Province, P.R. China; 3 Zhen Wei Department of Ultrasound, the third Hospital of Quzhou, 226 Baiyun North Avenue, Quzhou 324000, Zhejiang Province, P.R. China; 4 Zenghua Zhu Department of Radiology, the third Hospital of Quzhou, 226 Baiyun North Avenue, Quzhou 324000, Zhejiang Province, P.R. China

**Keywords:** Coronary slow flow, Doppler tissue imaging, Right ventricle, Retrospective analysis

## Abstract

**Objectives:**

To evaluate right ventricular diastolic function in patients with coronary slow flow phenomenon (CSFP) by using Doppler tissue imaging (DTI).

**Methods:**

CSFP patients diagnosed using coronary angiography from June 2019 to December 2020 at the third Hospital of Quzhou were retrospectively investigated, with a similar number of patients with normal coronary blood flow during the same period taken as the control group. Right ventricular systolic and diastolic function index was measured via DTI.

**Results:**

No differences were found between CSFP and control groups in terms of baseline data, RV end systolic diameter, RV end diastolic diameter, or RV ejection fraction. The peak velocity E in the early diastolic phase of the right ventricle was decreased in CSFP patients, while the peak velocity a in the late diastolic phase of the right ventricle was increased, resulting in a lower E / a ratio.

**Conclusions:**

Right ventricular diastolic function in patients with CSFP is decreased, and this can be identified using DTI. DTI has important applicative value for evaluating right ventricular diastolic function in patients with CSFP.

## INTRODUCTION

Coronary slow-flow phenomenon (CSFP) is an angiographic phenomenon where dye passage is slow in coronary angiography in the absence of significant arterial lesions or other causes.^1^ Despite considerable number of studies published since its discovery in 1972, the etiology and pathogenesis of CSFP remains unclear. CSFP is likely to lead to angina pectoris and myocardial infarction, possibly due to the impairment of coronary endothelial function, coronary microvascular lesions, early atherosclerosis, and myocardial infarction.^2^,^3^ Doppler tissue imaging (DTI) is often used to evaluate cardiac function in the clinic by measuring blood flow velocity under the annulus.^4^,^5^ This retrospective analysis investigated patients undergoing coronary angiography between June 2019 and December 2020. The purpose of the study was to determine the ability of Doppler tissue imaging technology to evaluate the right ventricular function of patients with CSFP, explore the characteristics of CSFP cardiac tissue ultrasound, and to provide a reference for the clinical prevention and treatment of CSFP.

## METHODS

The records of patients who underwent coronary angiography echocardiography in Quzhou Third Hospital from June 2019 to December 2020 due to chest tightness and chest pain, suspected of coronary heart disease, were retrospectively selected. Inclusion criteria was : echocardiography showing normal cardiac function (left ventricular ejection fraction > 50%). Exclusion criteria included presence of acute myocardial infarction, prior myocardial infarction, coronary aneurysm, coronary artery dilation, coronary spasm, cardiomyopathy, heart valve disease, myocardial bridge, or congenital heart disease; infectious diseases; liver and kidney diseases; presence of tumors; mild anemia (blood protein lower than 90g/L), severe cerebrovascular disease, or systemic immune system diseases. Based on these criteria, 45 patients were included in the CSFP group, while 42 patients with normal coronary artery blood flow comprised the control group. This research plan was approved by the medical ethics committee of our hospital (Approval no. LL21402, Date: 2021-06-29).

Patient information, including gender, age, BMI index, heart rate, and past medical history (including history of coronary heart disease, history of diabetes, history of hyperlipidemia, smoking history, and drinking history) were compiled and summarized. Laboratory examination results (fasting blood glucose, serum creatinine) were reviewed. Doppler tissue ultrasound examination and measurement indices (left ventricular end diastolic diameter, left ventricular end systolic diameter, interventricular septal thickness, left ventricular ejection fraction, right ventricular end diastolic diameter, left atrial diameter, and right atrial diameter) were collected. Right ventricular histologic ultrasound results (peak velocity of blood flow in early diastole, peak velocity of blood flow in late diastole, the ratio of peak velocity of blood flow in early and late diastole, isovolumic diastolic time, and Tei index) were examined.

CSFP was diagnosed via coronary angiography mainly using the corrected thrombolysis in myocardial infarction (TIMI) blood flow measurement frame method. The criteria for this method are as follows:^6^ the first frame is defined as when contrast agent completely or nearly completely fills the beginning of the coronary artery and touches the vessel wall and when the forward movement of the contrast agent can be seen. The final frame was defined as when the contrast medium reached the distal branch and specific anatomical landmarks were displayed. The normal range for coronary blood flow velocity was defined as 36.2±2.6 frames for the left anterior descending artery, 22.2±4.1 frames for the left circumflex artery, and 20.4±3.0 frames for the right coronary artery. If the TIMI frame count exceeded normal limits by more than two standard deviations, it was defined as CSFP.

Doppler tissue echocardiography and measurement methods were as follows: Live Five echocardiography (GE Healthcare) was used, with a probe frequency ranging between 2.5-4.0 mhz. The patient assumed left supine position for routine echocardiography. Doppler tissue imaging (DTI) was then used to measure right ventricular end systolic diameter (RVESD), right ventricular end diastolic diameter (RVEDD), and ejection fraction (EF). DTI sampling was taken under the tricuspid valve to obtain the forward bimodal laminar flow spectrum in the diastolic phase. The peak velocity (E) in early diastolic phase and peak velocity (a) in late diastolic phase were measured, and the E/A ratio was calculated.

Excel software (Microsoft) was used for data collection, and SPSS 22.0 software was used for data processing. Measurement data with normal distributions were summarized as Mean±SD and independent sample t tests were used to compare between groups. Counting data were expressed as n (%), with comparisons between groups performed using χ^2^ inspection. P < 0.05 indicated statistical significance.

## RESULTS

There were no significant differences in gender, age, BMI, heart rate, fasting blood glucose, serum creatinine levels and history of smoking, drinking, coronary heart disease, furosemide use and hyperlipidemia between CSFP and control groups (P>0.05, Table-I). No significant differences in cardiac Doppler ultrasound indices were noted between the two groups (>0.05, Table-II).

**Table-I:** comparison of basic data between the two groups [n(%), Mean±SD].

Index	CSFP group(n=45)	Control group(n=42)	χ²/t	P
Sex(Male/Female)	24/21	24/18	0.127	0.721
Age(year)	53.42±11.48	54.35±9.70	-0.409	0.684
BMI(kg/m^2^)	23.33±2.13	22.83±2.15	1.087	0.280
Heart rate(times/minute)	69.95±3.50	69.21±4.17	0.900	0.371
Smoke status(n)	19(42.22)	15(35.71)	0.386	0.534
Drinking status(n)	17(37.78)	12(28.57)	0.829	0.363
History of coronary heart disease(n)	7(15.56)	6(14.29)	0.028	0.868
Diabetes history(n)	6(13.33)	4(9.52)	0.310	0.578
History of hyperlipidemia(n)	4(8.89)	2(4.76)	0.576	0.448
Fasting blood glucose(mmol/L)	5.16±0.57	4.95±0.58	1.734	0.087
Serum creatinine(μmol/L)	84.57±7.30	85.62±7.79	0.644	0.522

**Table-II:** Comparison of cardiac Doppler ultrasound indexes between the two groups (Mean±SD).

Index	CSFP group(n=45)	Control group(n=42)	t	P
Left ventricular end diastolic diameter (mm)	48.53±1.57	49.07±1.85	-1.462	0.147
Left ventricular end systolic diameter (mm)	29.15±1.77	29.66±1.76	-1.349	0.181
Interventricular septal thickness (mm)	10.40±1.25	9.88±1.27	1.918	0.058
Left ventricular ejection fraction (%)	70.04±2.32	69.59±2.34	0.896	0.373
Right ventricular end diastolic diameter (mm)	28.91±1.70	28.57±1.72	0.923	0.358
Left atrial diameter (mm)	36.20±1.83	35.47±2.32	1.622	0.108
Right atrial diameter (mm)	32.97±3.03	33.47±3.18	-0.748	0.456

Right ventricular diastolic function, tricuspid annular velocity, and E peak on the blood flow spectrum were significantly decreased in the CSFP group (P< 0.05) while a peak was significantly increased (P<0.05). This resulted in a decreased E/A peak ratio (P<0.05). Tei index increased significantly in the CSFP group (Table-III).

**Table-III:** comparison of right ventricular systolic and diastolic function indexes of two groups by color Doppler ultrasound (Mean±SD).

Index	CSFP group(n=45)	Control group(n=42)	t	P
Peak velocity of blood flow in early diastole (cm/s)	57.31±4.47	71.85±3.96	-16.018	<0.001
Peak velocity of late diastolic blood flow (cm/s)	79.89±4.39	66.83±3.88	14.641	<0.001
Peak velocity ratio of early and late diastolic blood flow	0.72±0.07	1.08±0.09	-19.939	<0.001
Isovolumic relaxation time (MS)	78.97±3.47	65.04±1.97	22.774	<0.001
Tei index	0.50±0.07	0.40±0.07	6.117	<0.001

## DISCUSSION

CSFP is defined by the presence of a delayed perfusion in the distal vessel despite a lack of an obvious lesion in the coronary artery during coronary angiography.^7^,^8^ CSFP can lead to myocardial ischemia and heterogeneous changes in myocardial electrical activity that can be identified using body surface electrocardiograms. In particular, CSFP patients may present with fragmented QRS complexes.^9^,^10^ Moreover, increase in repolarization indices (P wave dispersion, QT dispersion) in various parts of the heart have been linked to CSFP occurance.^11^,^12^ In addition, right ventricular function can be evaluated using DTI.^13^-^15^ Indeed, researchers have previously used DTI-obtained right ventricular parameters to detect early lesions in diabetic patients.^16^ This study showed that DTI can accurately reflect RV systolic and diastolic function along the long axis and provide a basis for diagnosing CSFP patients. Specifically, when E/A is less than one, right ventricular diastolic function is normal.

The Tei index, first proposed in 1995, evaluates the systolic and diastolic function of the heart.^17^ It is the ratio of the sum of the isovolumic systolic interval and the isovolumic diastolic interval to the ejection time of the heart, and thus can directly reflect the overall function of the right ventricle and accurately evaluate right ventricular function. Indeed, studies have shown that among clinical (vascular congestion, functional grade disorder) and echocardiographic (index, right ventricular diameter, area fraction change, tricuspid annular plane systolic shift, S wave, Tei index) variables, Tei index was the only variable with high diagnostic ability. Here, we found that patients with CSFP had significantly longer isovolumic diastolic time and higher Tei index values compared with the control group.^18^

### Limitation of the study:

The main limitation of the study is the relatively small sample size and retrospective single-center nature. Larger multi-center studies that include different types of patients are merited.

## CONCLUSION

Although the sample size of this study is small, we demonstrated that right ventricular diastolic dysfunction in patients with CSFP may be an early manifestation of coronary slow flow. Therefore, accurate evaluation of right ventricular diastolic dysfunction may provide a reference point for early diagnosis of CSFP.

### Authors’ contributions:

**XZ:** Conceived and designed the study.

**XX & ZW:** Collected the data and performed the analysis.

**XZ:** Was involved in the writing of the manuscript, is responsible for integrity of the study

**ZZ:** Edited the manuscript and data analysis.

All authors have read and approved the final manuscript.
